# The AMPK Family Member Snf1 Protects *Saccharomyces cerevisiae* Cells upon Glutathione Oxidation

**DOI:** 10.1371/journal.pone.0058283

**Published:** 2013-03-05

**Authors:** Maria Pérez-Sampietro, Celia Casas, Enrique Herrero

**Affiliations:** Departament de Ciències Mèdiques Bàsiques, Universitat de Lleida, IRBLleida, Lleida, Spain; Texas A&M University, United States of America

## Abstract

The AMPK/Snf1 kinase has a central role in carbon metabolism homeostasis in *Saccharomyces cerevisiae*. In this study, we show that Snf1 activity, which requires phosphorylation of the Thr210 residue, is needed for protection against selenite toxicity. Such protection involves the Elm1 kinase, which acts upstream of Snf1 to activate it. Basal Snf1 activity is sufficient for the defense against selenite, although Snf1 Thr210 phosphorylation levels become increased at advanced treatment times, probably by inhibition of the Snf1 dephosphorylation function of the Reg1 phosphatase. Contrary to glucose deprivation, Snf1 remains cytosolic during selenite treatment, and the protective function of the kinase does not require its known nuclear effectors. Upon selenite treatment, a null *snf1* mutant displays higher levels of oxidized versus reduced glutathione compared to wild type cells, and its hypersensitivity to the agent is rescued by overexpression of the glutathione reductase gene *GLR1.* In the presence of agents such as diethyl maleate or diamide, which cause alterations in glutathione redox homeostasis by increasing the levels of oxidized glutathione, yeast cells also require Snf1 in an Elm1-dependent manner for growth. These observations demonstrate a role of Snf1 to protect yeast cells in situations where glutathione-dependent redox homeostasis is altered to a more oxidant intracellular environment and associates AMPK to responses against oxidative stress.

## Introduction

The AMP-activated protein kinase (AMPK) family is constituted by protein complexes that participate in metabolic stress responses addressed to maintain cellular ATP levels in eukaryotes [1]. Full activity of the catalytic α subunit of the AMPK complex requires phosphorylation of a T-loop threonine residue, as well as binding of the β and γ subunits. The only member of the AMPK family in *Saccharomyces cerevisiae* is Snf1, which plays a key role in adaptation of cells to glucose limitation and use of alternative carbon sources [2], [3]. Snf1 activity requires the participation of the regulatory γ subunit Snf4 and one of the three β subunits Gal83, Sip1 or Sip2. Upon glucose limitation, activation of Snf1 needs phosphorylation of the T-loop Thr210 residue. This is carried out by one of the three redundant kinases Sak1, Elm1 or Tos3 [4], [5]. Although Sak1 plays the most relevant role in such activation, only the absence of the three upstream Snf1-activating kinases causes complete inability for growth on carbon sources other than glucose, which indicates a partially redundant function of Sak1, Elm1 and Tos3 on Snf1 activation in glucose-limited conditions [4], [5]. On the other hand, the phosphorylation state of Thr210 is negatively regulated by the protein phosphatase 1 (PP1) complex. This is composed by the Glc7 catalytic subunit and the Reg1 regulatory subunit [6]. This complex seems to be the direct or indirect sensor of the cellular glucose status [7], [8]. Recently, PP2A-type phosphatases have also been implicated in regulating Snf1 phosphorylation and activity [8], [9]. Activated Snf1 in glucose limitation conditions regulates the expression of multiple genes, which are not necessarily related to carbon source metabolism [10]. Among the best-characterized nuclear protein targets of Snf1 are the Mig1 repressor and the Cat8, Sip4 and Adr1 transcriptional activators [3].

In addition to glucose limitation Snf1 also participates in the response of yeast cells to other environmental stresses. Thus, a null *snf1* mutant is hypersensitive to sodium and lithium or to hygromicin B [11]–[13], calcium excess [14], alkaline pH conditions [15], genotoxics such as hydroxyurea and methyl methane sulfonate [16], and cadmium [17]. Most of these stresses, when applied in normal glucose concentration conditions, cause phosphorylation of Snf1 Thr210, with Sak1 playing the major but not exclusive Snf1-activation role. Nevertheless, the levels of Snf1 activity required for responding to the above stresses are lower than those required for responding to glucose depletion [12]–[14], [16]. In some cases, such as the response to hydroxyurea or cadmium, the detectable phosphorylation levels of Snf1 upon stress do not rise over basal levels [14], [16] and a Snf1 mutant protein in which the Thr210 residue has been replaced with alanine is still able to protect against hygromicin B [11].

Selenium (Se) is an essential microelement in human cells present as selenocysteine in selenoproteins [18]. Among the latter, there are enzymes protecting against oxidation of macromolecules by reactive oxygen species. On the other hand, at high concentrations Se may be toxic because of the generation of oxidative stress conditions and DNA damage [18], [19]. *S. cerevisiae* is an adequate model to study the molecular basis of Se toxicity since this yeast lacks selenoproteins and therefore, Se is not required as growth factor. In *S. cerevisiae* the more toxic form of Se is selenide. This can be formed from other Se forms such as selenite [20], the predominant environmental form. In the presence of glucose, selenite enters the yeast cell through the high affinity phosphate transporter Pho84 in low phosphate conditions and through both Pho84 and the low affinity transporters Pho87/Pho90/Pho91 in high phosphate conditions [21]. Once inside the cell, selenite causes double-strand breaks, high mutagenicity rate, cell cycle arrest and protein hypercarbonylation which is indicative of extensive protein oxidative damage [22]–[24]. Overall, these effects may be indicative of intracellular oxidative stress caused by selenite. Transcriptomic studies [25] have demonstrated selenite-mediated upregulation of genes involved in high affinity iron uptake (whose expression is under the control of the Aft1 transcription factor) and in stress and protein degradation responses.

Based on the stress effects caused by selenite in yeast cells and on the protective role of Snf1 in defense against a diversity of stresses in addition to glucose depletion, this led us to explore the role of the Snf1 pathway in the response to selenite stress and in a broader perspective, in the response to changes in the redox state of the cell. Our results show that Snf1 activity is required to protect yeast cells against situations that decrease the ratio of reduced versus oxidized glutathione, including selenite treatment. We also demonstrate that such protective role of Snf1 takes place at the cytosol and does not correlate with extensive phosphorylation of Thr210 upon selenite addition. Overall, this study reveals a relationship between Snf1 kinase and redox regulation processes in yeast cells.

## Materials and Methods

### Strains and Plasmids

Strains employed in this study (W303 genetic background unless otherwise indicated) are listed in Table 1. Plasmids pWS93 and its derivatives pWS-Snf1, pWS-Snf1-T210A and pWS-Snf1-K84R, as well as pHA-Mig1 have been described [26]. Plasmid pYCp414 overexpresses *TRK1* and derives from vector pCM262 [27]. Plasmid pOV84 expresses a Snf1-GFP protein under the *SNF1* promoter [28]. Plasmid pMM1039 was obtained in this study by cloning the *GLR1* open reading frame under the control of the *tetO_7_* promoter in the centromeric vector pCM189 [29]. P1116 is a multicopy plasmid overexpressing *GLR1* under its own promoter [22].

**Table 1:** Strains employed in this study.

Strain	Genotype	Source and comments
W303-1A	*MAT*a *ura3-1ade2-1 leu2-3,112 trp1-1 his3-11,15 can1-1*	Wild type
Wsnf1	W303-1A *snf1::HIS3*	From Francisco Estruch
WΔ3	W303-1A *trk1::LEU2 trk2::HIS3*.	From Joaquin Ariño
MML348	W303-1A *aft1-Δ5::URA3*	From our laboratory
MML1304	W303-1A *pho84::natMX4*	This work
MML1370	W303-1A *sak1::natMX4*	This work
MML1387	W303-1A *sak1::natMX4 elm1::kanMX4*	This work, *elm1::kanNX4* mutation from YPDahl21
MML1389	W303-1A *sak1::natMX4 tos3::TRP1*	This work, *tos3::TRP1* mutation from YPDahl19
MML1390	W303-1A *elm1::kanMX4 tos3::TRP1*	This work, *elm1::kan MX4* and *tos3::TRP1* mutations from YPDahl21 and YPDah19
MML1392	W303-1A *sak1::natMX4 elm1::kanMX4 tos3::TRP1*	This work, *elm1::kan MX4* and *tos3::TRP1* mutations from YPDahl21 and YPDahl19
MML1396	W303-1A *sip4::kanMX4.*	This work
MML1401	W303-1A *snf1::HIS3 pho84::natMX4*	This work
MML1407	W303-1A *snf4::kanMX4*	This work
MML1408	W303-1A *mig1::kanMX4*	This work
MML1417	W303-1A *cat8::natMX4*	This work
MML1419	W303-1A *adr1::natMX4.*	This work
MML1442	W303-1A *reg1::URA3*	This work, from MCY3278 [6]
MML1445	W303-1A *sip1::natMX4 sip2::kanMX4*	This work
MML1447	W303-1A *snf1::HIS3 trk1::LEU2 trk2::HIS3*	This work, from WΔ3
MML1452	W303-1A *sip1::natMX4 gal83::HIS3*	This work, *gal83::HIS3* mutation from MSY558 [34]
MML1454	W303-1A *sip2::kanMX4 gal83::HIS3*	This work, *gal83::HIS3* mutation from MSY558 [34]
MML1459	W303-1A *sip1::natMX4 sip2::kanMX4 gal83::HIS3*	This work, *gal83::HIS3* mutation from MSY558 [34]
MML1724	W303-1A *snf1::HIS3 elm1::kanMX4*	This work
YPDahl19	W303-1A *tos3::TRP1*	From Stefan Hohmann [13]
YPDahl21	W303-1A *elm1::kanMX4*	From Stefan Hohmann [13]
DLY4033	*MAT*a *his3 ura3 lys2 trp1*	Wild type, from Gislene Pereira [38]
AKY516	*MAT*a *his3 ura3 lys2 trp1 ELM1-GFP::kanMX4*	Derived from DLY4033 [38]

### Growth Media and Culture Conditions

YPD (1% yeast extract, 2% peptone, 2% glucose) or synthetic SC medium were usually employed for *S. cerevisiae* cell growth. For glucose starvation conditions, concentration of glucose in the medium was 0.05%. YPGal and YPGly contain respectively 2% galactose or 3% glycerol instead of glucose. When required, YPD medium was supplemented with exogenous iron by addition of 90 µM BPS (phosphate buffered saline) plus 100 µM FeSO_4_ [40]. To control phosphate concentration in the growth medium, SD broth with 2% glucose w/o phosphate (Formedium) was employed as phosphate-depleted basal medium, to which KH_2_PO_4_ was added at 0.2 mM (low phosphate conditions) or 7.3 mM (normal phosphate conditions). In the former case, KCl was added up to 7.3 mM final concentration. Media were solidified with 2% agar. Sodium selenite (Sigma) was added at the concentration indicated in each case. Cells were grown at 30°C, with shaking in the case of liquid cultures.

### Genetic Methods

Standard protocols were used for DNA manipulations and transformation of yeast cells. Single null mutants were generated using the short-flanking homology approach after PCR amplification of the *natMX4* cassette and selection for nouseothricin resistance [30]. Disruptions were confirmed by PCR analysis. Null mutations in some genes were moved from the BY4741 or other genetic backgrounds to the W303 background after PCR amplification of the corresponding disruption cassette plus about 300 bp flanking genomic regions in the donor mutant, and subsequent transformation of the amplified fragment into wild type W303 cells. Multiple mutants were obtained by crossing the parental mutant strains, followed by diploid sporulation, tetrad analysis, and selection of the mutant combinations.

### Determination of Growth Sensitivities

Sensitivity to selenite was determined in plate growth assays by spotting serial 1∶10 dilutions of exponential cultures onto YPD or SC plates containing sodium selenite, and recording growth after 2 or 3 days of incubation at 30°C. Growth of several strains in liquid medium under parallel separate treatments was automatically recorded (optical density at 600 nm) at one-hour intervals during 24 hours, using individual 0.5 ml cultures in shaken microtiter plates sealed with oxygen-permeable plastic sheets, in a PowerWave XS (Biotek) apparatus at controlled temperature. Identical cell numbers (2×10^5^) were inoculated initially in each parallel culture.

### Northern Blot Analyses

RNA isolation and electrophoresis, probe labeling with digoxigenin, hybridization, and signal detection were done as described previously [29]. Gene probes were generated by PCR from genomic DNA, using oligonucleotides designed to amplify internal open reading frame sequences. *SNR19* mRNA was employed as loading control.

### Immunoblot Analyses of Snf1 Phosphorylation at Thr210

From cell samples obtained at the indicated times in each experiment, protein extracts were prepared by the heat inactivation/alkaline treatment method [31]. They were separated by SDS-PAGE and analyzed by immunoblotting with anti-phospho-Thr172-AMPK (Cell Signaling Technology) at 1∶1,000 dilution. Membranes were reprobed to determine total Snf1 levels with rabbit polyclonal anti-Snf1 antibodies [12] at 1∶1,000 dilution.

### Microscopy Methods

GFP-tagged proteins were visualized with an Olimpus BZ51 fluorescence microscope, after nuclear staining of cell samples with Hoesch (5 µg ml^−1^, 6 min). U-MNUA2 and U-MNUA3 filters were employed respectively for Hoesch and GFP staining. Immunofluorescence experiments to localize HA-tagged Mig1 were done with 3F10 rat anti-HA (Roche) and Alexa488 goat anti-rat (Molecular Probes) and parallel nuclear staining with DAPI.

### Analytical Methods

Cellular concentrations of oxidized and reduced glutathione were determined by using the Ellman’s reagent method in culture samples quenched by 5-sulfosalicylic acid [32]. Cell concentration and cell volume values were respectively determined in formaldehyde-fixed and non-fixed samples using a Coulter Z2 analyzer, and were employed to calculate glutathione concentrations. Glucose-6-phosphate intracellular concentration was determined by electrospray mass spectrometry from 20 mg cells (dry weight). Sample quenching and extraction was made using tricine buffer pH 7.4 [33]. The methanol/water phase was evaporated in vacuum and resuspended in 100 µl of water/ethanol (50/50, v/v). An Agilent 1290 LC system coupled to an ESI-Q-TOF MS/MS 6520 instrument (Agilent Technologies) was used, employing a column with 1.8 µM particle size. MassHunter Qualitative Analysis Software (Agilent Technologies) was used for integration and extraction of peak intensities. The m/z value for quantification was 259.0224 [M-H]^−^. Levels were adjusted to internal standard (phenylalanine-C13, m/z 165.076 [M-H]^−^).

## Results

### Snf1 Activity Protects Against Selenite Toxicity

**Figure 1:**
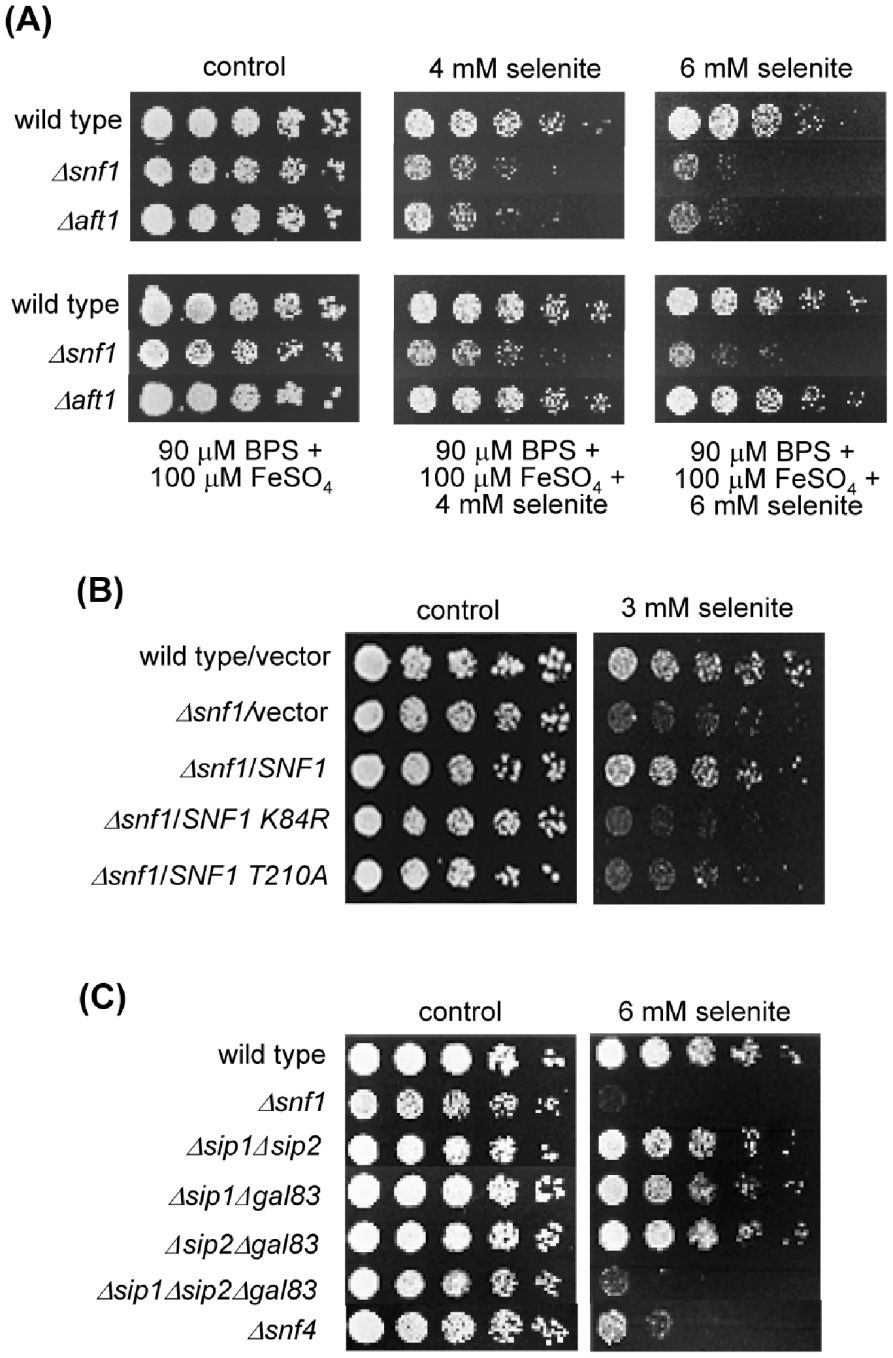
Snf1 activity is required for protection against selenite. (**A**) Growth assays of serial dilutions of the respective strains on YPD medium with the indicated additions. Growth was recorded after 48 hours at 30°C. Strains employed: wild type (W303-1A), *Δsnf1* (Wsnf1) and *Δaft1* (MML348). (**B**) Growth assays of serial dilutions of the following strains, plated on SC medium with sodium selenite: wild type (W303-1A) and *Δsnf1* (Wsnf1) cells transformed with vector pWS93, and *Δsnf1* cells transformed with pWS-Snf1, pWS-Snf1-T210A and pWS-Snf1-K84R. Growth was recorded after 3 days at 30°C. (**C**) As in (A), with the following strains in addition to wild type and *Δsnf1*: *Δsip1Δsip2* (MML1445), *Δsip1Δgal83* (MML1452), *Δsip2Δgal83* (MML1454), *Δsip1Δsip2Δgal83* (MML1459) and *Δsnf4* (MML1407).

We initially determined the sensitivity of a *S. cerevisiae Δsnf1* null mutant to selenite stress. The mutant was more sensitive to the agent than wild type cells (Figure 1A). Given that selenite treatment of yeast cells induces expression of Aft1-dependent genes of the high affinity mechanism for iron uptake [25], in parallel we determined the selenite sensitivity of a *Δaft1* mutant. This was also hypersensitive to the agent, but while its hypersensitivity to selenite was rescued in iron-repletion conditions, this was not the case for the *Δsnf1* mutant (Figure 1A). The observation supports an iron-independent protective role of the Snf1 kinase against selenite toxicity.

Next, we determined whether Snf1 activity is required for protection after selenite treatment, by employing yeast mutants expressing two mutated forms of Snf1. The *snf1-K84R* mutant expresses a Snf1 form which lacks a Lys84 residue required for ATP binding, and consequently has a very low kinase activity, while the *snf1-T210A* mutant expresses a Snf1 form lacking the Thr210 residue subjected to activating phosphorylation [3]–[5]. None of the two Snf1 mutant forms expressed from a plasmid rescued the selenite hypersensitivity of the *Δsnf1* null strain, compared with a control wild type Snf1 form (Figure 1B). This therefore confirms that the Snf1 kinase activity is needed either for the response or for recovery from selenite stress.

. Snf1 activity requires one of the three β subunits Sip1, Sip2 or Gal83, which confer substrate specificity to the complex [28], [34]. Under glucose deprivation, Gal83 internalizes into the nucleus together with Snf1, while Sip1 becomes vacuole-associated and Sip2 remains dispersed at the cytosol [28], [35]. Although in these glucose-minus conditions Gal83 seems to be the main Snf1 activator, the other two β subunits may have redundant roles as shown from the growth phenotypes of the respective single and double mutants in glucose-deprived medium [34]. Using a similar approach, we explored the participation of the three β subunits in protection against selenite. The strain lacking all three β subunits was as sensitive to selenite as the *Δsnf1* mutant, while the strains expressing only one of the three subunits displayed a wild type phenotype (Figure 1C). Therefore, any of the Sip1, Sip2 and Gal83 proteins alone may activate Snf1 with full efficiency against selenite toxicity. Consistently with the role of the Snf4 γ subunit on Snf1, a *Δsnf4* strain was also hypersensitive to selenite, although it displayed a slightly milder phenotype than mutants in the α or β subunits (Figure 1C).

### Elm1 Kinase Plays an Important Role in Protection Against Selenite Toxicity

**Figure 2:**
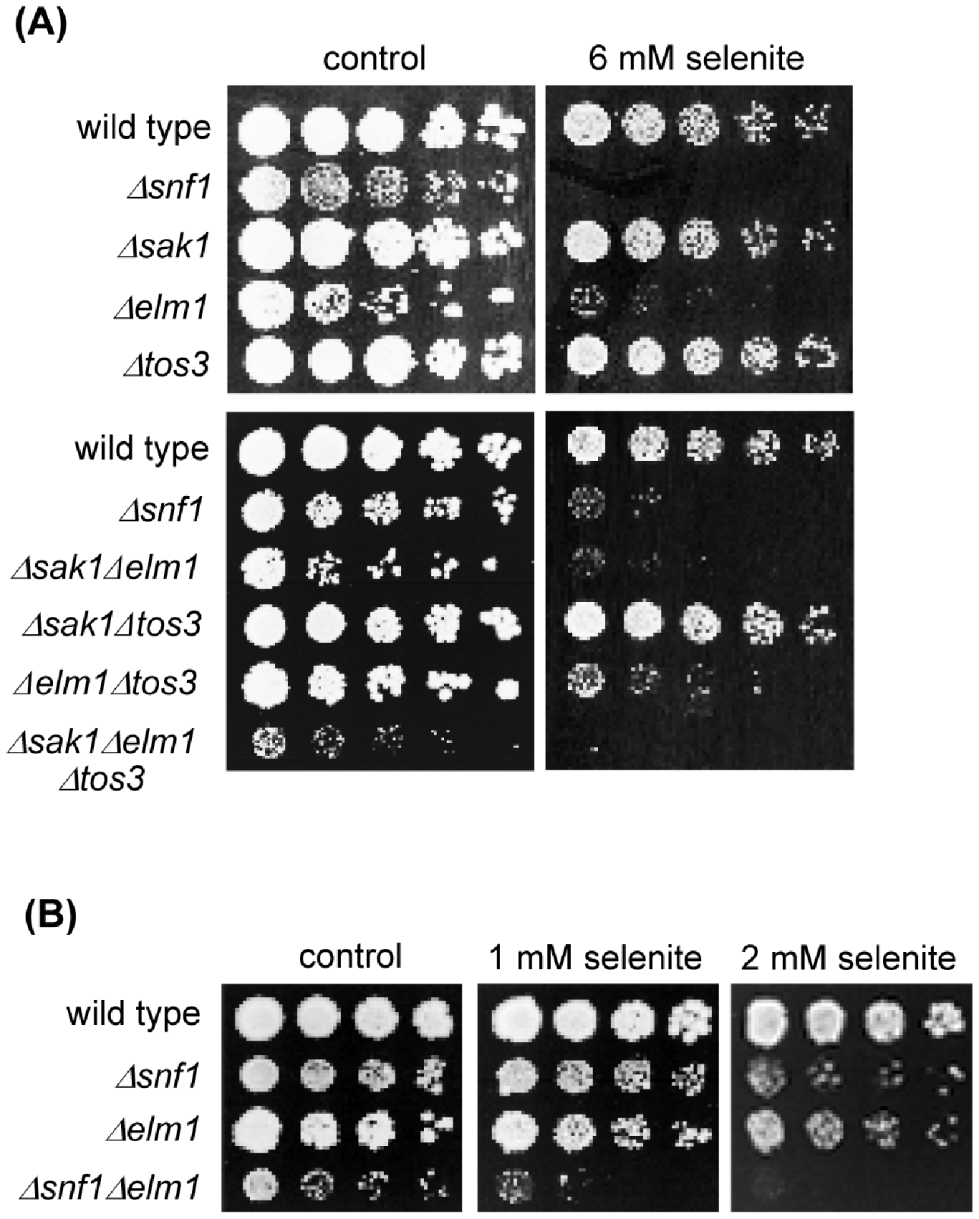
Protection against selenite preferentially requires the Elm1 kinase. (**A**) Growth assays of serial dilutions of the following strains on YPD medium with sodium selenite: wild type (W303-1A), *Δsnf1* (Wsnf1), *Δsak1* (MML1370), *Δelm1* (YPDahl21), *Δtos3* (YPDahl19), *Δsak1Δelm1* (MML1387), *Δsak1Δtos3* (MML1389), *Δelm1Δtos3* (MML1390) and *Δsak1Δelm1Δtos3* (MML1392). (**B**) As in (A) with the strains: wild type, *Δsnf1*, *Δelm1* and *Δsnf1Δelm1* (MML1724).

Three upstream kinases (Sak1, Elm1 and Tos3) participate in Thr210 phosphorylation and activation of Snf1 in response to glucose depletion and other stresses. They act redundantly, although in many cases (for instance in glucose-minus conditions) Sak1 plays the most relevant role [3]. We tested the selenite sensitivity of the individual null mutants in each of the three Snf1 kinases (Figure 2A). Only the *Δelm1* mutant was more sensitive than the wild type strain, displaying almost the same sensitivity levels as the *Δsnf1* mutant, which suggests an important role for Elm1 in the selenite stress response. This was confirmed when the sensitivity of the double mutants was tested (Figure 2A). Cells expressing only Tos3 were as sensitive to selenite as the *Δsnf1* mutant or the mutant lacking all three upstream kinases, indicating that Tos3 plays no protective role at all. Cells expressing only Sak1 displayed an intermediate sensitivity phenotype, while cells expressing only Elm1 had the same sensitivity to selenite as wild type cells. Therefore, Elm1 is sufficient for protection against this agent, although in its absence Sak1 would perform some protection.

Elm1 and Snf1 carry out partially independent parallel roles in the response to sodium stress [13], contrary to the response to glucose deprivation in which both kinases participate in the same pathway. We therefore determined whether the former was also the case in the protective response to selenite. With this objective, we studied the sensitivity to the agent in the double *Δsnf1Δelm1* mutant compared to the single *Δsnf1* and *Δelm1* mutants. The double mutant displayed additive sensitivity (Figure 2B), indicating that besides its activating role on Snf1, Elm1 carries out protective functions against selenite which are Snf1-independent.

### Snf1 Remains at the Cytosol Upon Selenite Treatment

**Figure 3:**
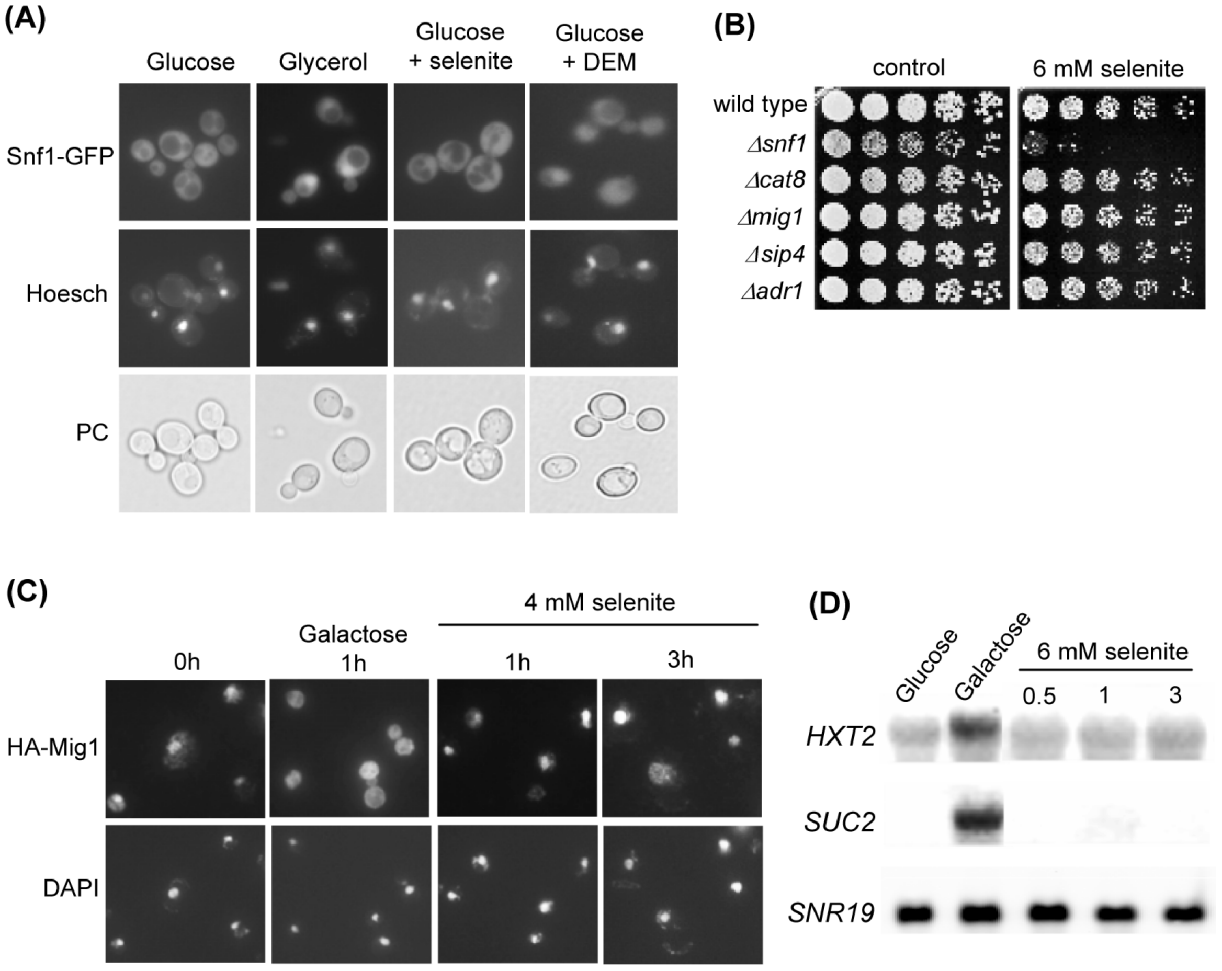
Protection against selenite toxicity does not require activity of the known nuclear effectors of Snf1. (**A**) Localization of Snf1 upon different treatments. The Snf1-GFP protein expressed in pOV84-transformed wild type cells was visualized by fluorescence microscopy in cells growing in SC medium without treatment (Glucose) or after 2 hours treatment with 4 mM sodium selenite or 1 mM DEM. In parallel, cell samples were shifted to YPGly and observed one hour later (Glycerol). Prior to observations, samples were stained with Hoesch for nuclei localization. The corresponding phase contrast fields (PC) are shown. (**B**) Growth assays of serial dilutions of the following strains on YPD medium with sodium selenite: wild type (W303-1A), *Δsnf1* (Wsnf1), *Δcat8* (MML1417), *Δmig1* (MML1408), *Δsip4* (MML1396) and *Δadr1* (MML1419). (**C**) Localization of HA-Mig1 upon different treatments. Cells transformed with pHA-Mig1 were grown in SC medium and treated with selenite for the indicated times or shifted to medium with 2% galactose instead of glucose. Cells were observed by immunofluorescence experiments with anti-HA antibodies, with parallel nuclear staining with DAPI. (**D**) Northern blot expression analysis of the indicated genes in wild type (W303-1A) cells growing in YPD medium without (Glucose) or with selenite for the indicated times (hours), or in YPGal medium (Galactose) for 1 hour. *SNR19* was employed as loading control. The same blotted membrane was successively hybridized with the three probes after extensive washings.

A fraction of Snf1 molecules localize to the nucleus upon glucose depletion, under regulation by Gal83 [28]. Alkaline stress also causes Snf1 nuclear localization [12]. Using a Snf1-GFP construction, we determined its location after application of a selenite stress. Snf1 remained at the cytosol during the entire period of treatment, contrary to cells that had been shifted to glycerol medium (Figure 3A). These results suggested that protection against selenite does not require the nuclear pool of activated Snf1, and are in accordance with the main role of Elm1 as activator of Snf1 upon selenite stress. In fact, Elm1 has been characterized as a bud neck-associated kinase playing important roles in septin organization and cytokinesis [36], [37] and in the spindle position checkpoint [38], [39]. Using a functional Elm1-GFP form [38], we could determine that Elm1 protein remains at the bud neck in selenite-treated cells (Figure S1).

Other experiments confirmed the nucleus-independent function of Snf1 in selenite stress signaling. First, mutants in nuclear effectors of Snf1 such as Cat8, Mig1, Sip4 or Adr1 [3], [10] were as sensitive to selenite as wild type cells (Figure 3B), supporting that none of them is involved in protection against the agent. Next, we employed Mig1 localization as reporter of Snf1-mediated signal transduction to the nucleus. Thus, upon glucose depletion Snf1 phosphorylates Mig1 promoting its export from the nucleus and consequent derepression of glucose-repressed genes [26]. While shifting the cells from glucose to galactose-based medium caused rapid exit of Mig1 from the nucleus, extensive nuclear Mig1 staining was still observed after 3 hours of selenite treatment (Figure 3C). In parallel, we determined the mRNA levels of two genes, *HXT2* and *SUC2,* whose expression is repressed by Mig1 in glucose medium and activated in a Snf1-dependent manner upon shifting to alternative carbon sources. In contrast to control cells after 1 hour in galactose medium, no detectable derepression of the expression of *HXT2* and *SUC2* was observed even after 3 hours of treatment with selenite (Figure 3D). Overall, these experiments support that the selenite-induced signal does not regulate the activity of nuclear effectors of Snf1 such as Mig1, and that Snf1 plays its protective role at the cytosol.

### Phosphorylation of Snf1 does not Correlate with Protection Against Selenite Toxicity

**Figure 4:**
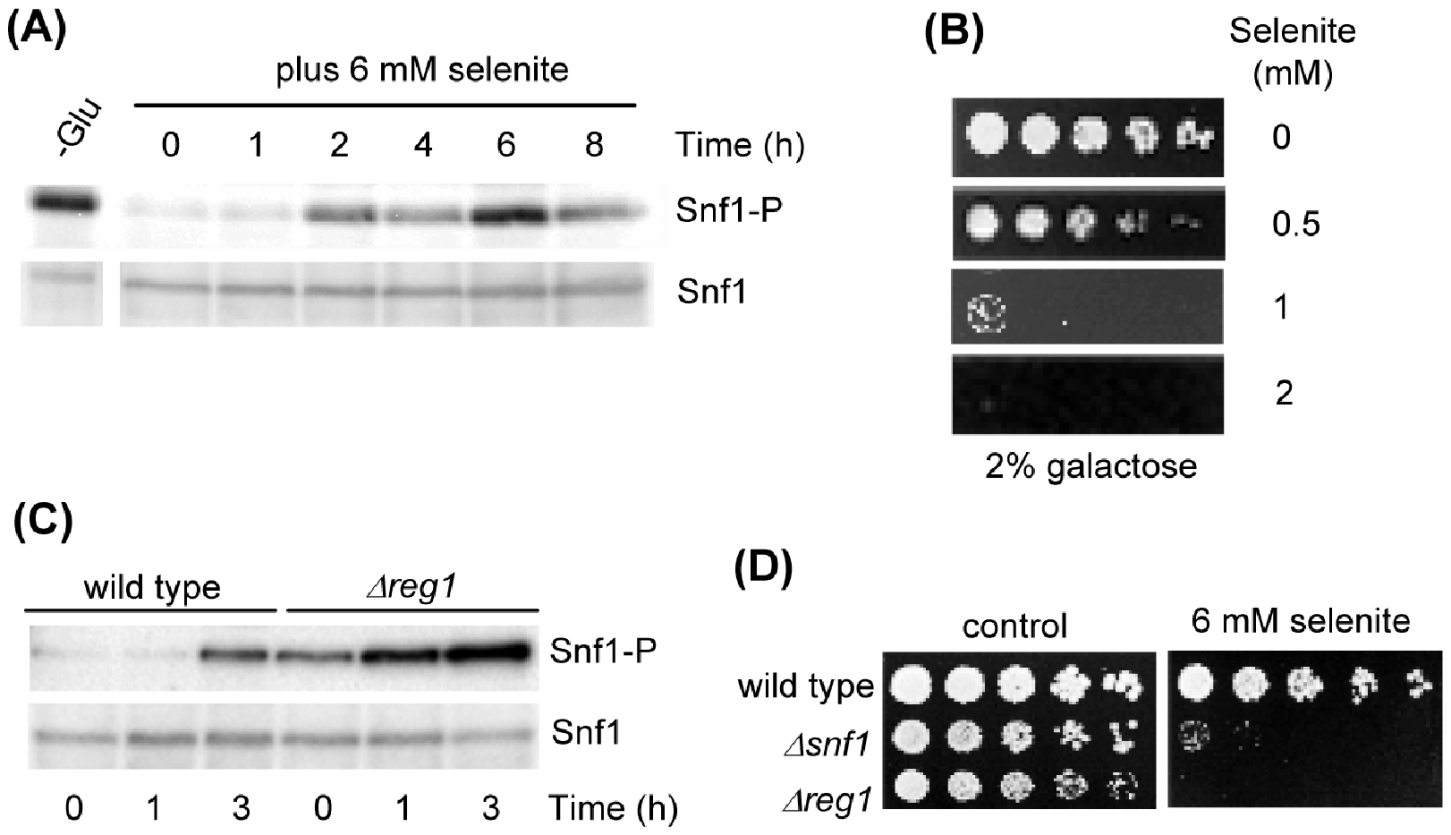
Phosphorylation levels of Snf1 at Thr210 do not correlate with protection against selenite treatment. (**A**) Western blot analysis of phosphorylated Snf1 at Thr210 with anti-phospho-Thr172-AMPK (upper panel). Blots were rehybridized with anti-Snf1 antibodies for total Snf1 (lower panel). Samples were obtained from wild type (W303-1A) exponential cultures in YPD treated with sodium selenite for the indicated times. Control samples were run from YPD-grown wild type cells that were shifted for 1 hour to YPGly (-Glu). (**B**) Growth assays of serial dilutions of wild type (W303-1A) cells in YPGal medium with the indicated concentrations of selenite. (**C**) As in (A) with samples from wild type and *Δreg1* (MML1442) cells. (**D**) Growth assays of serial culture dilutions of wild type (W303-1A), *Δsnf1* (Wsnf1) and *Δreg1* (MML1442) strains on YPD medium with selenite.

We next studied whether Snf1 becomes phosphorylated at Thr210 upon selenite addition. Western blot analyses using an antibody specifically recognizing the Snf1 form phosphorylated at the T-loop Thr residue demonstrated a moderate phosphorylation of Thr210 after selenite addition, which was only manifested after 2 hours of treatment and at later times (Figure 4A). This Snf1 phosphorylation therefore occurred in growth medium with normal glucose levels (2% concentration), and did not reach the Thr210 phosphorylation levels observed upon glucose depletion. Lower selenite concentrations provoked a weaker and less sustained response (data not shown). The delayed Snf1 phosphorylation response upon selenite treatment could reflect indirect effects not necessarily related to the protective role of Snf1 against the agent. Additional observations by us confirmed that phosphorylation of Snf1 is not required for protection against selenite. Thus, in spite of the fact that Snf1 becomes phosphorylated at Thr210 when yeast cells are grown in carbon sources alternative to glucose, the sensitivity of the yeast cells in front of selenite was increased when growing with galactose (Figure 4B) or glycerol [24] as only carbon sources compared to cells growing in glucose-based medium. We also determined the sensitivity to selenite of a null *Δreg1* mutant that displays constitutive Snf1 phosphorylation in glucose medium [7], [8]. In this mutant, selenite caused additional phosphorylation of Snf1 over the basal levels at earlier times than in wild type cells (Figure 4C). However, the *Δreg1* mutant did not display higher resistance to selenite than wild type cells, and in fact was as sensitive to the agent as *Δsnf1* cells (Figure 4D). This may point to interfering effects of selenite on Reg1 and probably other phosphatases (see Discussion). In any case, the above results together indicate that although Snf1 activity mediated by Thr210 phosphorylation is required for protection against selenite toxicity, increased phosphorylation over the basal levels does not cause further protection.

### The Selenite Toxic Effects are not Due to Glucose Deprivation by the Agent

The delayed phosphorylation of Snf1 upon selenite treatment seems to indicate that this agent does not cause immediate glucose deprivation effects and therefore discards this as an explanation for the growth sensitivity effects of the mutant. That selenite does not induce expression of *HXT2* or *SUC2* also argues against occurrence of glucose deprivation effects caused by the agent. However, we further addressed this point using several approaches. First, we asked whether the hypersensitivity of the *Δsnf1* mutant could be caused by selenite effects at the cell surface not able to be counteracted in the absence of Snf1 function. Thus, plasma membrane depolarization induced by selenite could inhibit glucose uptake and consequently activate Snf1. The high affinity potassium transport system formed by Trk1 and Trk2 is a main determinant of the yeast plasma membrane electrochemical potential [40]. In case that selenite provokes membrane depolarization, hyperpolarized *Δtrk1Δtrk2* mutants would be less sensitive to selenite and the absence of Trk1 and Trk2 would at least partially rescue the selenite sensitivity of *Δsnf1* cells [11]. Similarly, overexpression of *TRK1* depolarizes the plasma membrane [27] and this would exacerbate the selenite sensitivity of *Δsnf1* cells. However, these hypothesis were not confirmed (Figure S2A), which argues against selenite effects on plasma membrane polarization. Next, we determined whether growing cells in medium with higher glucose concentration than normal rescued selenite toxicity. Contrary to other stresses such as alkaline treatment [15], the selenite sensitivity of *Δsnf1* compared to wild type cells was not affected by growing cells in 5% glucose (Figure S2B). Finally, we determined intracellular glucose-6-phosphate levels upon selenite treatment to determine possible effects of the agent on glucose uptake. Wild type cells treated during 4 hours did not show a decrease of the levels of glucose-6-phosphate (Figure S2C). On the contrary, intracellular levels of this metabolite increased at initial times to regain the initial levels at 4 hours. This initial increase may be related to the selenite-mediated transcriptional upregulation of carbohydrate synthesis genes described in Ref. 25. In *Δsnf1* cells, a similar kinetics of the evolution of intracellular glucose-6-phosphate was observed, although concentrations of the metabolite were lower than in wild type cells at all treatment times as well as in untreated cultures (Figure S2C), indicating that this is a selenite-independent effect. In summary, the experiments argue against an intracellular glucose-depletion effect by selenite, and support that Snf1 phosphorylation at advanced treatment times is not due to glucose deprivation.

### Toxicity by Selenite Requires Entry of the Agent into the Cells

**Figure 5:**
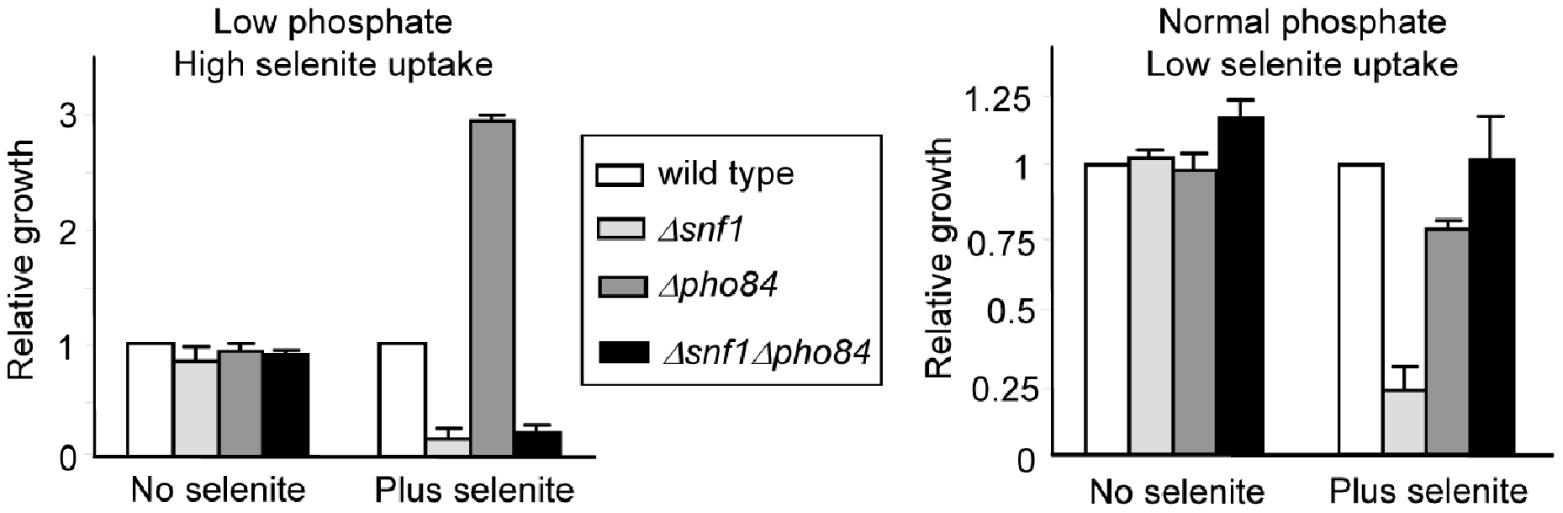
Effect of selenite on growth in low or normal phosphate conditions. Relative growth of wild type (W303-1A), *Δsnf1* (Wsnf1), *Δpho84* (MML1304) and *Δsnf1Δpho84* (MML1401) cells in low or normal phosphate medium without or with 3 mM selenite. Growth in shaken microtiter plates was automatically recorded and the growth values reached by each strain after 24 hours were made relative to the growth of wild type cells, which was given the unit value for each growth condition considered. The mean of three independent experiments (± s.d.) is represented. Note that different scales of the *y-*axis are employed in both panels.

To further address the causes of the hypersensitivity of *Δsnf1* mutant cells to selenite, we studied whether the mutant was still hypersensitive to selenite when entry of this agent into the cells was inhibited. In fermentative growth conditions such as those employed in the current study, selenite enters *S. cerevisiae* cells through the high affinity phosphate transporters Pho84 in low phosphate medium, and through both the high affinity and the low affinity transporters in normal phosphate medium [21]. We took advantage of the fact that expression of *PHO89*, which codes for the alternative high affinity transporter Pho89, is induced by selenite and is under the control of Snf1 in both low and normal phosphate medium (Figure S3). Therefore, in *Δsnf1Δpho84* cells in low phosphate conditions no expression of the high affinity phosphate transporters occurs and phosphate transport may be compromised. This situation would be exacerbated in the presence of 3 mM selenite, which competes with the low phosphate amounts for entrance through the poorly operational low affinity transport system [21]. The resulting phosphate starvation conditions would explain the poor growth displayed by the *Δsnf1Δpho84* cells in the presence of selenite under low phosphate conditions (Figure 5). However, in normal phosphate conditions the susceptibility of the double mutant to selenite is similar to wild type cells (Figure 5). That is, in the absence of the Pho84/Pho89 high affinity transporter and when the high phosphate concentration (7.3 mM) is advantageously competing with 3 mM selenite for the low affinity transporter [21], then selenite toxicity is decreased in a *Δsnf1* background. Altogether, these observations support that the severe effects of selenite on *Δsnf1* cells require entry of the agent into the cells.

### Sensitivity of Snf1-deficient Cells to Selenite is Related to Alterations in Glutathione Metabolism

The tripeptide glutathione (L-γ-glutamyl-L-cysteinylglycine, GSH and GSSG respectively in its reduced and oxidized forms) is an essential thiol redox regulator [41]. Selenite causes depletion of GSH in yeast [20] as well as in other organisms [18]. Accordingly, overexpression of the *S. cerevisiae* glutathione reductase gene (*GLR1*) rescues selenite sensitivity [22]. In addition, in yeast cells selenite induces expression of *GLR1* and another key gene for GSH metabolism, *GSH1* (for L-γ-glutamyl-L-cysteine synthetase) [25]. Alteration of GSH metabolism could therefore explain the differential toxic effects of selenite on *Δsnf1* cells, and we explored this possibility.

**Figure 6:**
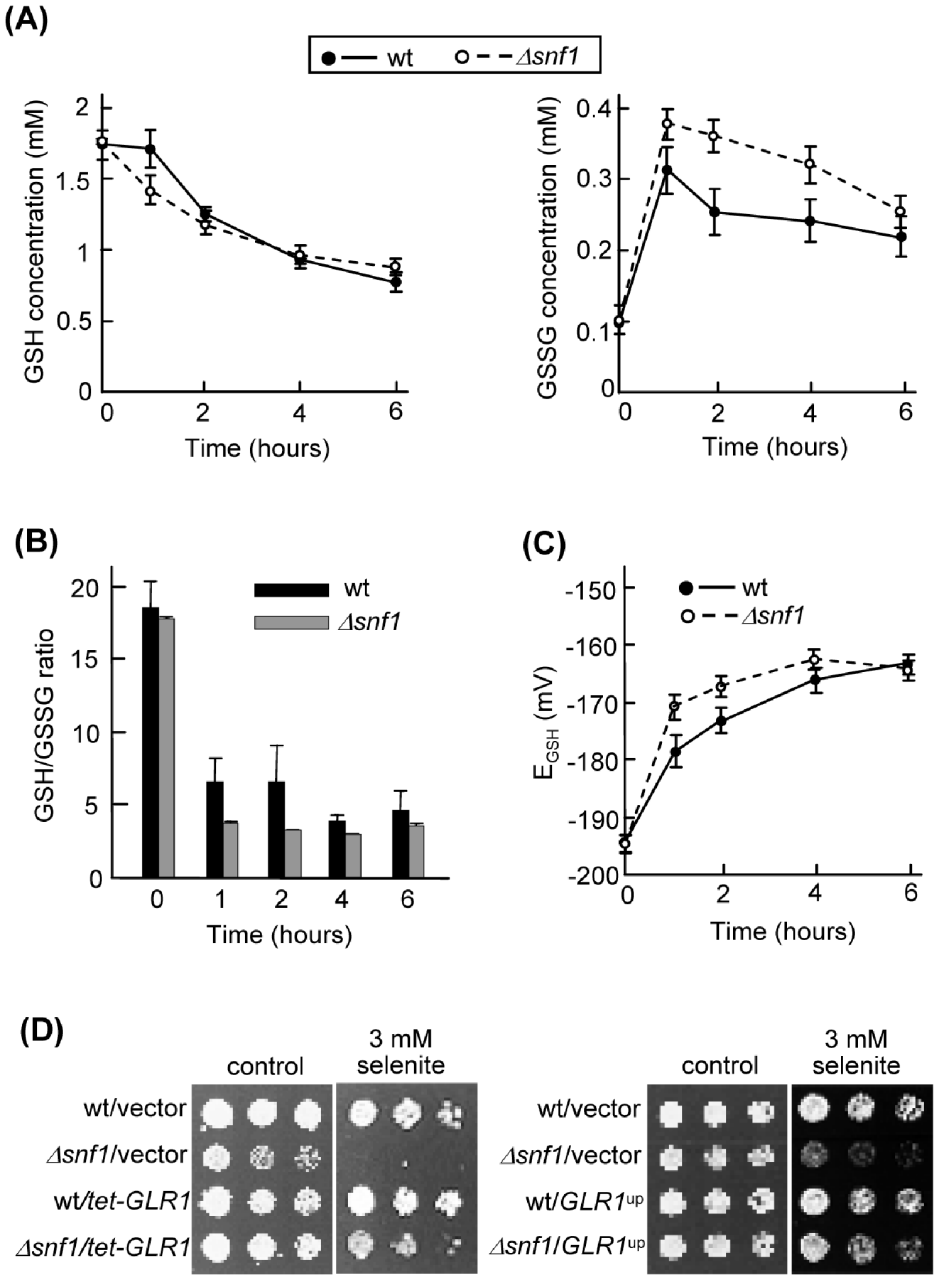
The ratio of reduced vs oxidized glutathione is altered upon selenite treatment. (**A**) Intracellular concentration of GSH (left) and GSSG (right) in cells treated with 2 mM sodium selenite for the indicated times. Wild type (W303-1A, continuous lines) and mutant *Δsnf1* (Wsnf1, dashed lines) cells were grown in SC medium. Values (± s.d.) are the mean of three independent experiments. (**B**) GSH/GSSG ratio as determined from the concentration values shown in part (A). (**C**) GSH redox potential (E_GSH_) in wild type (continuous lines) and mutant *Δsnf1* (dashed lines) cells treated with selenite. E_GSH_ was calculated from the GSH and GSSG concentration values in each of the three experiments indicated in part (A), using the Nernst equation for the GSH/GSSG pair. The mean (± s.d.) is represented. (**D**) Growth assays in SC medium of serial dilutions of the strains indicated in part (A) transformed with vector pCM189 or its derivative pMM1039 (*tetO-GLR1*), or of the same strains transformed with the multicopy vector YEplac195 or its derivative P1116 overexpressing *GLR1* (right panels).

First, we observed that selenite causes a more pronounced transitory induction of *GLR1* and *GSH1* expression in *Δsnf1* than in wild type cells, which may be indicative of more intense alteration in glutathione pools in the mutant (Figure S4). In order to confirm this, we measured GSH and GSSG intracellular concentration upon selenite treatment in the wild type and mutant strains (Figure 6A). The GSH pool decreased in both strains during the next 6 hours after selenite addition, following similar kinetics. On the contrary, GSSG accumulated at significantly higher levels in *Δsnf1* cells, particularly during the initial 4 hours of treatment. Consequently, the mutant cells exhibited a lower GSH/GSSG ratio during initial treatment times (Figure 6B). When the GSH redox potential (*E*_GSH_) was calculated from the GSH/GSSG concentrations, it began with almost identical values in untreated cultures of both strains but increased ∼16 mV in wild type cells and ∼24 mV in mutant cells after one hour of selenite treatment, and only returned to similar values for both strains after 6 hours of treatment (Figure 6C). These results confirmed that glutathione homeostasis is more dramatically altered by selenite in the absence of Snf1. To correlate the effects on the GSH/GSSG ratio with the growth effects of selenite, we overexpressed *GLR1* from a multicopy plasmid or from the *tetO_7_* promoter in both wild type and *Δsnf1* cells. With both overexpression strategies the relative hypersensitivity of *Δsnf1* cells in selenite plates was significantly rescued (Figure 6D). This therefore supported the explanation that the inhibitory effects of selenite on *Δsnf1* cells are caused by the accumulation of GSSG relative to GSH.

### Snf1 Kinase Activity Protects Against Agents Causing GSH Oxidation

**Figure 7:**
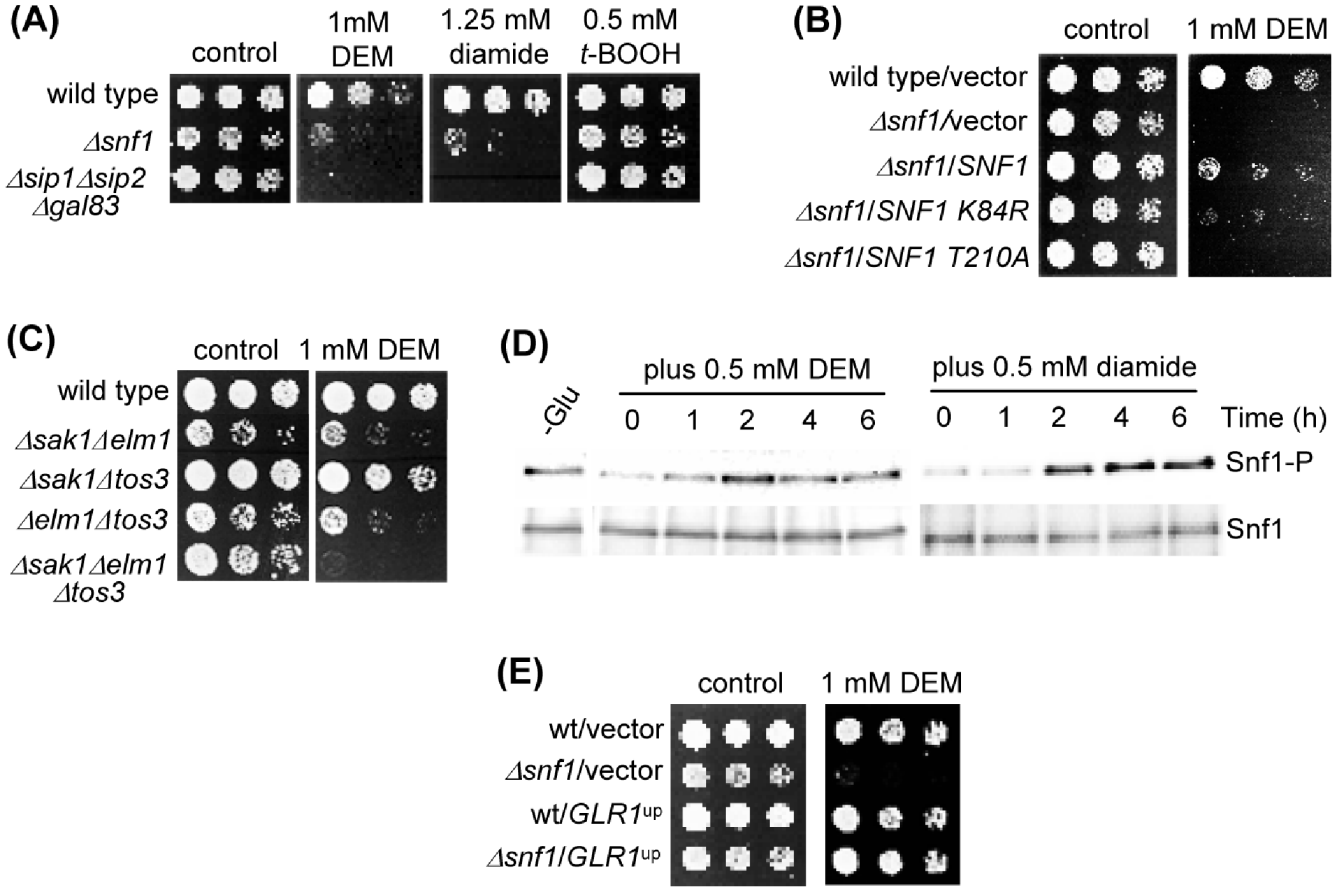
Snf1 is required for protection against glutathione-oxidizing agents. (**A**) Growth assays of serial dilutions of the following strains on YPD medium with DEM, diamide or *t*-BOOH: wild type (W303-1A), *Δsnf1* (Wsnf1) and *Δsip1Δsip2Δgal83* (MML1459). (**B**) Growth assays of serial dilutions of the following strains, plated on SC medium with DEM: wild type (W303-1A) and *Δsnf1* (Wsnf1) cells transformed with vector pWS93, and *Δsnf1* cells transformed with pWS-Snf1, pWS-Snf1-T210A and pWS-Snf1-K84R. (**C**) Growth assays of serial dilutions of the following strains on YPD medium with DEM: wild type (W303-1A), *Δsak1Δelm1* (MML1387), *Δsak1Δtos3* (MML1389), *Δelm1Δtos3* (MML1390) and *Δsak1Δelm1Δtos3* (MML1392). (**D**) Western blot analysis of Thr210-phosphorylated Snf1 (Snf1-P) and of total Snf1. The same membrane was successively hybridized with the corresponding antibodies. Samples were from wild type (W303-1A) exponential cultures in YPD treated with DEM or diamide. Control samples were from YPD-grown wild type cells shifted for 1 hour to YPGly (-Glu). (**E**) Growth assays of wild type (W303-1A) and *Δsnf1* (Wsnf1) cells transformed with multicopy vector YEplac195 or its derivative P1116 overexpressing *GLR1*, in SC medium with DEM.

The previous observations pointed to a general role of Snf1 in the defense against glutathione oxidation. To test this hypothesis, we carried out several experiments. First, we employed other agents that provoke changes in GSH homeostasis by oxidizing this molecule, such as diethyl maleate (DEM) [42] or diamide [43]. Absence of the Snf1 activity either in cells lacking Snf1 or the three β subunit components caused hypersensitivity to both DEM and diamide (Figure 7A). This is in contrast with the normal sensitivity of the mutants to *t-*butyl hydroperoxide (*t-*BOOH), which discards a general protective role of Snf1 activity in oxidative stress conditions. To confirm that Snf1 activity is required to counteract the effects of GSH-oxidizing agents, we showed that the *snf1-K84R* and *snf1-T210A* mutations do not complement the DEM-sensitive phenotype of a *Δsnf1* mutant, in contrast to the wild type form (Figure 7B). As in the case of selenite, the presence of the upstream kinase Elm1 alone was sufficient to confer wild type levels of DEM sensitivity to yeast cells (Figure 7C), and DEM did not provoke migration of Snf1 into the nucleus (Figure 3). Also as with selenite, both DEM and diamide caused phosphorylation at Snf1 Thr210 but only after 2 hours of treatment (Figure 7D). Finally, we confirmed that overexpression of *GLR1* protected the mutant against DEM toxicity (Figure 7E).

Summarizing, our results showed that Snf1 activity has a general protective role in yeast cells in situations that lead to glutathione oxidation such as the presence of selenite, DEM or diamide.

## Discussion

AMPK/Snf1 responds to metabolic stress in yeast cells, its kinase activity being required for adaptation to glucose limitation and growth in alternative carbon sources [3]. Snf1 also plays a defense function against a number of environmental stresses (see Introduction), although this role does not always require additional activation of Snf1 over basal levels [14], [16]. In contrast, oxidative stress by peroxide also activates Snf1, but in this case the kinase does not seem to participate in protection of yeast cells against peroxide toxicity [12,16, and this work]. In the present study we have shown that Snf1 activity is required for defense of *S. cerevisiae* cells upon selenite treatment. Although we observed phosphorylation of Snf1 Thr210 at advanced treatment times, the basal activity of Snf1 is sufficient to protect against the agent, and nutritionally or genetically provoked situations where Snf1 becomes permanently phosphorylated at Thr210 do not provide additional selenite resistance. Since Snf1 phosphorylation levels are mainly regulated by the activity of Reg1 [7], [8], the late phosphorylation of Snf1 provoked by selenite could be due to inactivation of Reg1 (and alternative PP1- or PP2A-type phosphatases) by the agent. Inhibitory modulation of the activity of PP1 phosphatases by selenite has been described in human cells [44]. The proposition that in yeast cells selenite could affect the activity of Reg1 and other phosphatases is supported by the observations that: (i) a *Δreg1* mutant is also hypersensitive to the agent (this work), and (ii) a *Δppz1* mutant is also unable to grow in the presence of selenite (our unpublished results). Ppz1 is a phosphatase with large homology to PP1-type phosphatases whose roles in the regulation of cation homeostasis and other yeast cell processes have been characterized [40].

We have shown that selenite toxicity and the requirement of Snf1 for protection against it is not related to plasma membrane depolarization effects and/or glucose depletion, and that such protection requires the entry of the agent into the cell mostly through the high affinity mechanism of phosphate uptake, which is the main mediator of selenite entry in glucose-grown yeast cells [21]. Once into the cell, selenite does not provoke migration of Snf1 into the nucleus, in contrast to the activation of Snf1 by glucose deprivation and the consequent response. Thus, at least the upstream steps of the Snf1-mediated response to selenite seem to occur entirely at the cytoplasm. In addition, none of the characterized transcription regulators participating in the response to metabolic stress by glucose deprivation (Cat8, Mig1, Sip4 or Adr1) appear to be individually important in protection against selenite. Our results are reminiscent of earlier studies [4], [13] which showed that exposure of cells to high sodium led to increased phosphorylation of Snf1 without phosphorylation of Mig1 and without induction of glucose-controlled genes. Noteworthy, Snf1 does not only regulate nuclear targets, but also modulates the function of cytosolic proteins, such as the arrestin-related protein Rod1, which coordinates endocytosis of alternative carbon source transporters in response to glucose presence in the medium [45]. Thus, Snf1 may have a wider range of targets (both cytosolic and nuclear) than previously expected. The important function of Elm1 during the selenite response seems surprising. In addition to its overlapping role with Sak1 and Tos3 in Snf1 activation during glucose depletion, Elm1 is required for the organization of the septin network at the bud neck [36], [37] and coordinates the spindle position checkpoint through activation of the Kin4 kinase [38], [39]. Different regions of the protein molecule could participate in such diversity of Elm1 functions [38], [39], [46]. Selenite does not provoke significant alterations in the yeast cell cycle or in cell morphogenesis [22], [24], neither delocalizes Elm1 from the bud neck (Figure S1). This argues against, although it does not entirely prove, the participation of the bud neck-associated Elm1 pool in the selenite response, and supports additional functions for cytoplasmic Elm1 molecules. In any case, Elm1 has a role in defense against selenite which is in part independent of the Snf1 role, a similar situation to the participation of Snf1 and Elm1 in protection against salt stress [13].

How selenite signaling is related to Snf1 activity? Selenite provokes a reduction of GSH and an increase of GSSG in yeast cells [20], [47]. Such decrease of the ratio GSH/GSSG is exacerbated in cells lacking Snf1 up to four hours of treatment. Differences between wild type and mutant cells were mainly due to a relative increase of GSSG in the latter rather than to differences in the GSH pool between both strains. Overexpression of the *GLR1* glutathione reductase gene protects yeast cells against the toxic effects of selenite [22]. Along this line, overexpression of *GLR1* in *Δsnf1* cells allows growth of the mutant in the presence of selenite up to similar levels as the wild type. This fact points to the changes (compared to wild type cells) of the redox potential of the GSH/GSSG pair as the cause of the hypersensitivity of *Δsnf1* cells to selenite. Accordingly, yeast cells lacking Snf1 are also hypersensitive to agents such as DEM or diamide, which increase the GSSG pools relative to GSH. In addition, overexpression of *GLR1* also allows growth of *Δsnf1* cells at the same level as wild type cells in the presence of DEM. Therefore, the relationship between selenite toxicity and cell protection mediated by Snf1 reflects a more general role of this kinase in sensing and responding to intracellular redox changes due to an increase of oxidized glutathione. Again, both DEM and diamide provoke a late phosphorylation of Snf1, which indicates that the possible alteration of the modulatory mechanisms of Snf1 phosphorylation is not circumscribed to selenite, but extends to a broader range of agents acting on intracellular redox homeostasis.

The signaling role of Elm1/Snf1 in inducing a protective response to a sudden increase of the GSSG/GSH ratio does not seem to involve previous functions assigned to Elm1 and Snf1, and as discussed above such role would not require the already characterized nuclear effectors of Snf1. Recent studies point to functions of Snf1 other than those previously characterized as metabolic regulator. Thus, analysis of the yeast kinase-protein interactome using protein microarrays has revealed common targets between Snf1 and the Akl1 kinase involved in cytoskeletal functions [48]. Another study integrating transcriptomic, proteomic and metabolomic data using a systemic a revealed that in addition to modulating other metabolic processes, Snf1 could be a regulator of redox homeostasis through the activity of Yap1, a transcription factor of genes participating in the oxidative stress response [49]. In human cell lines, hydrogen peroxide activates AMPK as part of a protective signaling mechanism mediated by mTORC1 [50]. In another study with human colon cancer cells, selenate provoked a late activation of AMPK through ROS formation and this AMPK activation was essential to inhibit cell proliferation by downregulating the COX2-mediated pathway [51]. An additional work with human cell lines also demonstrated activation of AMPK by redox changes in the α and β subunits induced by hydrogen peroxide [52]. The present study in yeast cells suggests that changing the redox pair GSH/GSSG to a more oxidant status induces a pathway mediated by Snf1 resulting in cell protection against oxidant conditions, for instance by rescuing the function of some redox-sensitive molecule essential for cell proliferation. Alternatively, although not in contradiction with the previous hypothesis, Snf1 could activate cell functions cooperating with the GSH-reducing system in maintenance of the GSH redox homeostasis in the presence of selenite and other oxidants. Based on all those observations, the participation of AMPK activity in regulation of cellular redox homeostasis could be a general property in eukaryotic cells. Further studies are required to characterize the downstream effectors of Snf1/AMPK that are important in the response to changes in the GSH/GSSG ratio, as well as to determine how the complex senses such changes.

## Supporting Information

Figure S1 **Location of Elm1 protein upon selenite treatment.** Strain AKY516 expressing *ELM1-GFP* derivative was grown in YPD medium with 4 mM selenite for the indicated times. Cells were immediately visualized by fluorescence and phase contrast microscopy using an Olympus BZ51 apparatus.
(TIF)

Figure S2 Selenite sensitivity is not associated to alterations in plasma membrane polarization or intracellular glucose deprivation. (A, left) Growth assays of serial dilutions of the following strains on YPD medium with selenite: wild type (W303-1A), *Δsnf1* (Wsnf1), *Δtrk1Δtrk2* (W3) and *Δsnf1Δtrk1Δtrk2* (MML1447). The medium was supplemented with 100 mM NaCl to improve growth of *Δtrk* cells. (A, right) Growth assays of serial dilutions of wild type (W303-1A) and *Δsnf1* (Wsnf1) cells transformed with vector pCM262 or its derivative pYCp414 overexpressing *TRK1*, in SC medium with selenite. (B) Growth assays of serial dilutions of wild type (W303-1A) and *Δsnf1* (Wsnf1) cells in YPD medium with the indicated concentrations of glucose plus selenite. (C) Relative amount of glucose-6-phosphate per cell. Samples were taken from wild type (W303-1A) or *Δsnf1* (Wsnf1) cells growing exponentially in YPD medium and treated with selenite for the indicated times. Values (± s.d., mean of six experiments) were made relative to the unit value corresponding to untreated wild type cells (absolute concentration of the relative unit value: 4.8×10^−11^ nmols per cell).
(TIF)

Figure S3 **Expression of** ***PHO89*** **is induced by selenite under the control of Snf1.** Northern blot expression analysis of the indicated genes in wild type (W303-1A), *Δsnf1* (Wsnf1), *Δpho84* (MML1304) and *Δsnf1Δpho84* (MML1401) cells in low or normal phosphate cultures treated with 3 mM selenite. *SNR19* was employed as loading control.
(TIF)

Figure S4 **Northern blot expression analysis of** ***GSH1*** **and** ***GLR1*** **in selenite-treated cells.** Exponential cultures of wild type (W303-1A) and *Δsnf1* (Wsnf1) cells in YPD medium were treated with 6 mM sodium selenite for the indicated times. *SNR19* was employed as loading control.
(TIF)
