# Supplementary figures and images for: The AMPK Family Member Snf1 Protects Saccharomyces cerevisiae Cells upon Glutathione Oxidation
Source: PLoS One. 2013 Mar 5;8(3):e58283. doi: 10.1371/journal.pone.0058283 (PMC3589272; doi:10.1371/journal.pone.0058283)

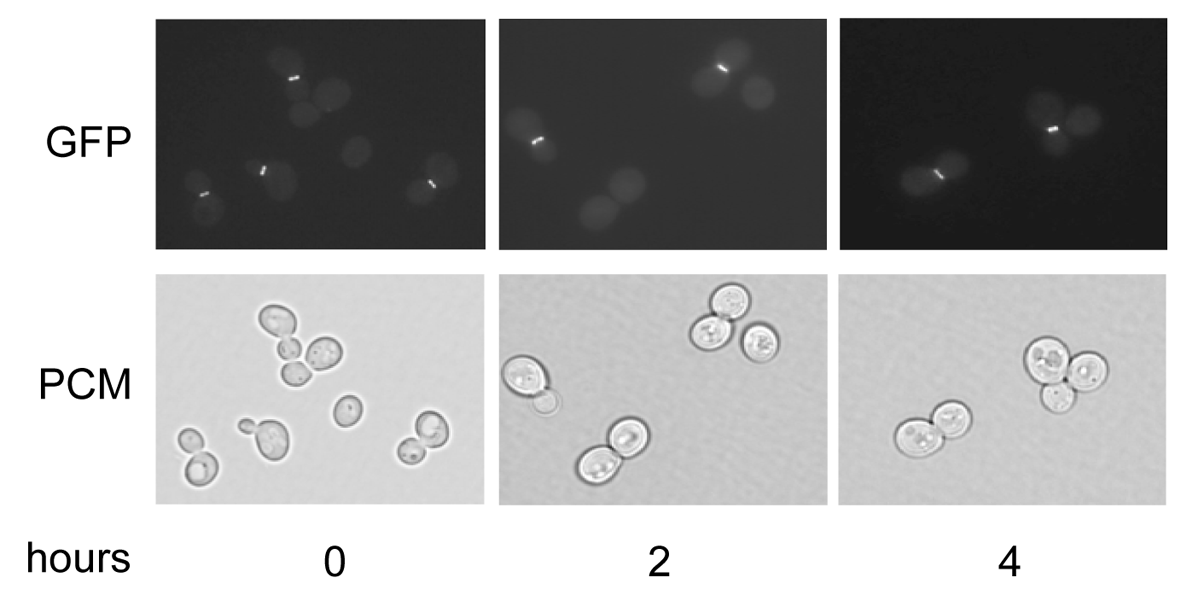

Supplement: Figure S1 — Location of Elm1 protein upon selenite treatment. Strain AKY516 expressing ELM1-GFP derivative was grown in YPD medium with 4 mM selenite for the indicated times. Cells were immediately visualized by fluorescence and phase contrast microscopy using an Olympus BZ51 apparatus. (TIF) [file pone.0058283.s001.tif]

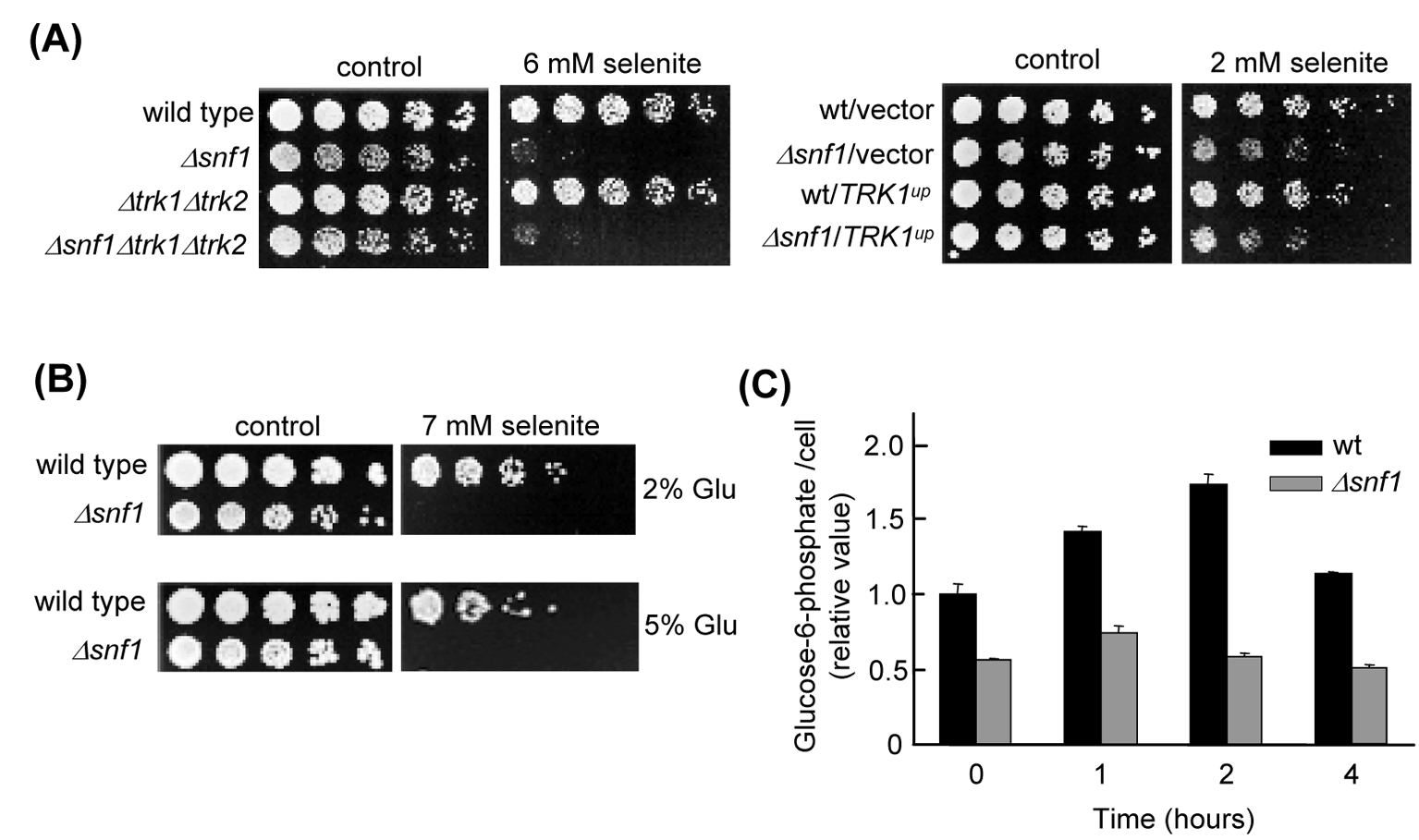

Supplement: Figure S2 — Selenite sensitivity is not associated to alterations in plasma membrane polarization or intracellular glucose deprivation. (A, left) Growth assays of serial dilutions of the following strains on YPD medium with selenite: wild type (W303-1A), Δsnf1 (Wsnf1), Δtrk1Δtrk2 (W3) and Δsnf1Δtrk1Δtrk2 (MML1447). The medium was supplemented with 100 mM NaCl to improve growth of Δtrk cells. (A, right) Growth assays of serial dilutions of wild type (W303-1A) and Δsnf1 (Wsnf1) cells transformed with vector pCM262 or its derivative pYCp414 overexpressing TRK1, in SC medium with selenite. (B) Growth assays of serial dilutions of wild type (W303-1A) and Δsnf1 (Wsnf1) cells in YPD medium with the indicated concentrations of glucose plus selenite. (C) Relative amount of glucose-6-phosphate per cell. Samples were taken from wild type (W303-1A) or Δsnf1 (Wsnf1) cells growing exponentially in YPD medium and treated with selenite for the indicated times. Values (± s.d., mean of six experiments) were made relative to the unit value corresponding to untreated wild type cells (absolute concentration of the relative unit value: 4.8×10^−11^ nmols per cell). (TIF) [file pone.0058283.s002.tif]

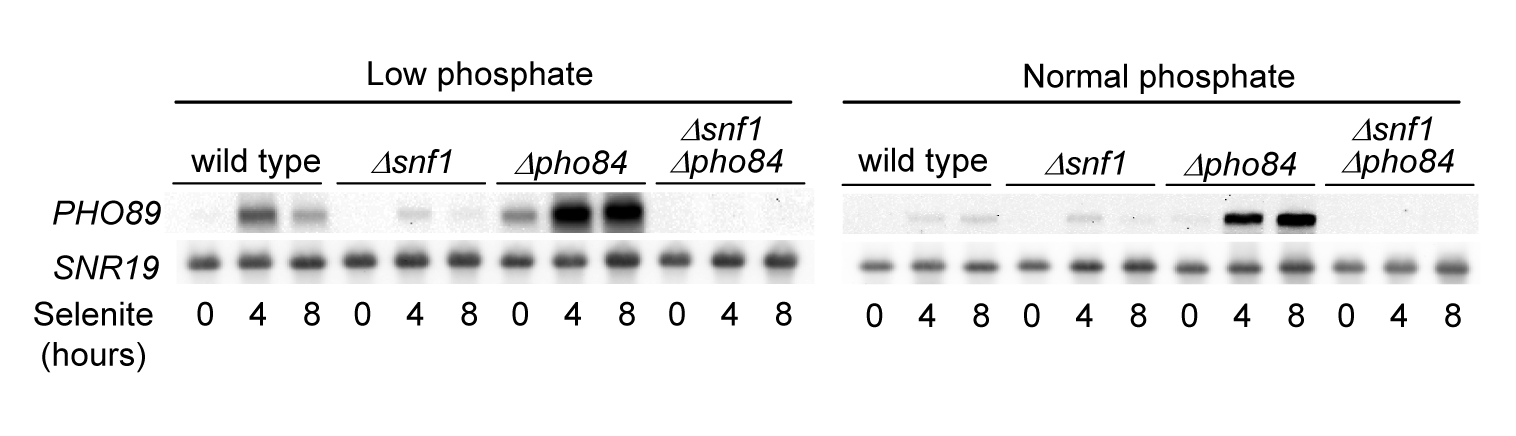

Supplement: Figure S3 — Expression of PHO89 is induced by selenite under the control of Snf1. Northern blot expression analysis of the indicated genes in wild type (W303-1A), Δsnf1 (Wsnf1), Δpho84 (MML1304) and Δsnf1Δpho84 (MML1401) cells in low or normal phosphate cultures treated with 3 mM selenite. SNR19 was employed as loading control. (TIF) [file pone.0058283.s003.tif]

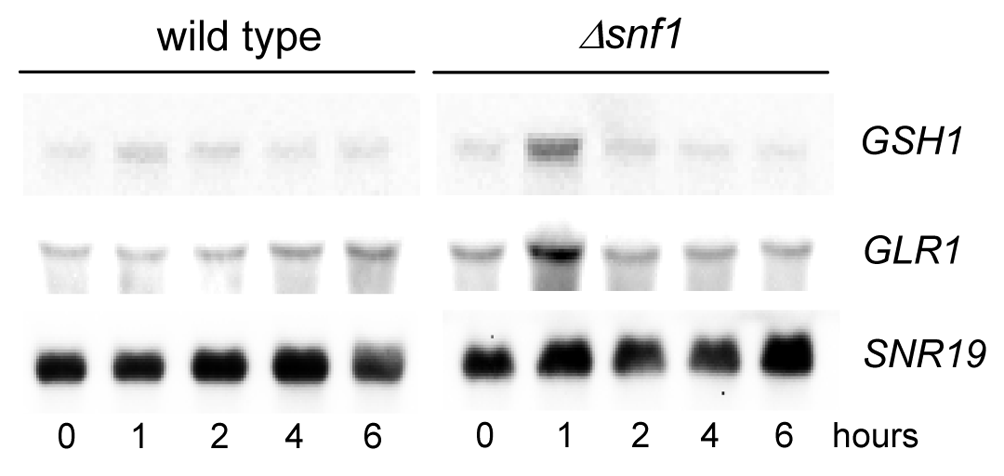

Supplement: Figure S4 — Northern blot expression analysis of GSH1 and GLR1 in selenite-treated cells. Exponential cultures of wild type (W303-1A) and Δsnf1 (Wsnf1) cells in YPD medium were treated with 6 mM sodium selenite for the indicated times. SNR19 was employed as loading control. (TIF) [file pone.0058283.s004.tif]
